# The Medical Profession in Parliament

**Published:** 1918-10-05

**Authors:** 


### THE MEDICAL PROFESSION IN PARLIAMENT.

### Sir Henry Morris, Bart., F.R.C.S., on Its Representation.

A largely-attended meeting of members of the medical profession was held on Tuesday, October 1, in the Steinway Hall, Wigmore Street, to consider the necessity, in the public interest, of having medical men to represent constituencies in Parliament.

The chair was occupied by Sir Henry Morris, Bart., F.R.C.S., and he was supported by the Right Hon. Christopher Addison, M.P. (Minister of Reconstruction), Sir William Watson Cheyne, Bart., C.B., M.P., Sir St. Clair Thomson, Sir Thomas Horder, Colonel Woodwark, A.M.S., Lieut.-Col. C. H. Fagge, Dr. Arthur Latham, Dr. C Briscoe, Mr. Ernest Clarke, Mr. P. Lockhart Mummery, Sir Arbuthnot Lane, Dr. H. Chapple, M.P., Dr. Leonard Williams, and Dr. Moreland McCrea. During the opening address of the Chairman and Dr. Addison's speech, as well as the brief and business-like proposals of Dr. Latham and Sir W. Watson Cheyne, the meeting was attentively decorous, but thereafter feeling ran somewhat high, and the multiplicity of amendments and modifications of the suggested resolutions promised a deadlock on more than one occasion. The courteous firmness of Sir Henry Morris, however, brought the meeting through, and he well merited the encomiums accorded to him at the end. The lack of union, which was given by more than one speaker as the cause of the small representative work done by the profession in this way hitherto, was thus illustrated in a striking manner.

The Chairman said that after the war the medical profession, Parliamentary constituencies, and Parliament would none of them be the same as they were before the war. With regard to the medical profession there were changes in prospect, both in reference to the organisation and the social relations, and the new Representation of the People Bill had altered the electorate considerably, the number of voters having been increased from eight millions to about eighteen millions, of whom about six millions were enfranchised women. The initiators of this meeting had not been animated by the thought of opposing any association, union, or committee which might be working for a similar object. The idea had been threefold: (1) To elect an executive committee at a meeting of the whole of the profession, not representative of any one section of it, however large; (2) the committee to select suitable members of the profession who were willing to stand for Parliament; and (3) to choose them from among consultants, general practitioners, representatives of Boards of Health, or other scientists. Two subjects of prime importance which such members would require to deal with were medical education and medical research, and a third was the application of scientific discovery for the betterment of the public health. But medical education and medical research must be endowed by State aid; it could no longer be self-supporting. He asked, Was the House of Commons, as at present constituted—consisting largely of lawyers, landlords, mineowners, shipowners, successful commercial men, and a few educationists—the body to decide such questions as ought to be settled in regard to public education? It was high time for the profession to take steps to show its importance to the State and to the public through the voice of competent and reliable members of the profession. Each medical candidate for Parliament should possess learning, knowledge, humanity, and probity; he should be acquainted with some form of public official life, be able to express himself clearly and forcibly, be an expert in some department of his profession, and give evidence of his intentions to act strictly for the public good, and more for the interests of the race than for the material advantage of his own profession. They did not want men who would hug delusions as if they were moral principles, nor fads as if they were ideals; neither were men required who would take part in political intrigues under the impression that they were democratic reforms. Hitherto the profession had never had any political standing in this country, but that state of things should not continue. Doctors had not the time individually to court constituencies, but the representation in Parliament must be brought about through the medical corporations, which included practically every practitioner of medicine.

The Right Hon. Christopher Addison, M.P., opened with a reference to the surrender of Bulgaria and the brighter dawn for Serbia, whose people had shown such a heroic and indomitable spirit, an example to mankind throughout the ages. The present meeting was, he said, full of significance, and was a sign of the times. After the war there would still be need for each of us to make the very best of what was in us. And the great need was knowledge: not prejudice, not guesswork, nor preconceived notions, but knowledge. And in public affairs this knowledge would require to be fortified by courage, and a determination which would not weaken with time. In the many matters which concerned the nation's well-being there was a growing need for the assistance, experience, and guidance of men who had the advantage of medical knowledge and practice. When speaking of politics he begged his hearers to rise above party limitations, above that monster known as the caucus. In these days of stern reality the nation would have but little patience with that kind of thing. The medical man in Parliament should be, first and foremost, a representative of the constituents. It was bad to witness the performances of the professional axe-grinder; in the end that process would fail and the public interest would suffer. By promoting the public interest, the professional interest would be safe enough. There would be great and increasing need for the presence of medical men of the right type and training in Parliament. To them the House of Commons would be a most tolerant and kindly place, for it put sincerity and earnestness of purpose, if coupled with common sense, in the forefront of its regard. But it would not be dictated to, and on occasion it could be swift enough in its actions. The idle or incompetent person was a hindrance, and might be a danger, in time of war, and that would be equally so in the period of reconstruction: there would be no room for waste, either of human or material things. Everything must be done to remove the handicap imposed on the nation by the numerous C3 class, which was only an expression, in adult life, of other armies of the same kind which were coming on from the crib. Here was a great opportunity for directed medical effort. The Prime Minister had said we could not expect an A1 Empire with a C3 population; and it would have been true to add that one could not expect an A1 population out of C3 homes, nor out of C3 habits or workshops or working conditions. Until there was a systematic health policy commensurate with the urgent national need, we should not cease to be the victims of sporadic, disjointed, and often futile effort. The recent deliberations of representative medical bodies had resulted in a report, which had now been passed on to the War Cabinet, and he would be disappointed if proposals for this needed reform were not presented to Parliament in the very near future. The Ministry of Health would not be a Health Service; it would be its duty to develop health administration throughout the country, and it was an essential part of that project that there should be connected with the Ministry certain consultative councils, of which one must be medical, and their duty would be to advise upon proposals, to make suggestions according to the best knowledge available. There was a great measure of agreement as to what was required at the beginning, and in the Budget of 1914 a million pounds was provided for improved Health Services. They had in mind at that time improved nursing, midwifery, laboratory facilities, and the provision of clinics—what Sir Bertrand Dawson well called "team work." Those who were concerned with the business of Government would learn a good deal from medical men, and it was conceivable that medical men would learn something also. The medical profession had now a unique opportunity before it, and it was one of the attributes of our race that in time of need men and women came forward and proved equal to the occasion. He moved "That in the interests of the national health it is essential that the considered views of the medical profession should be voiced by medical men to represent constituencies in the House of Commons."

Dr. Arthur Latham seconded in a short speech, in which he asked whether doctors realised that they were the trustees of the national health, and that no one could take their place. In the past the profession had no political power because it had not been united. Possibly such union would come some day, and it was hoped this meeting would be a step in the right direction.

Mr. E. B. Turner supported, and spoke of the awakened interest in health matters which he had experienced in speaking throughout the country concerning venereal disease.

Sir W. Watson Cheyne, M.P., pointed out that there would not be enough medical men in Parliament to influence vote by numbers; it must be done by judicial and well-informed utterance. And there was great need that the medical representatives should be united on the questions coming before Parliament; divided counsels would destroy the whole effect of special knowledge.

Colonel Kempson thought expert advice was wanted even more in the constituencies than in Parliament.

The resolution was carried.

Dr. E. F. White moved: "That a Representative Committee be appointed for the purpose of (i) nominating medical men as being suitable to voice the opinion of the profession in the House of Commons on matters affecting the national health; (ii) taking such steps as may be possible to further the election of those nominated to the House of Commons, and that this Committee report to a subsequent general meeting of the profession."

Mr. W. P. Lockhart Mummery seconded. Considerable discussion, led by Colonel Kempson, ensued, the chief point of which was that the meeting was asked to pledge itself to names with which it had only just been confronted and the possessors of which were but imperfectly known to many. An amendment was proposed and seconded to the following effect: "That a Representative Committee be appointed for the purpose of taking such steps as may be possible to further the election of medical men to the House of Commons."

Dr. Brackenbury, in supporting it, referred to the work of the Committee of the British Medical Association, which had been in existence six months. There must not only be union but funds.

Colonel Smith, a Sheriff of the City of London, considered that this method of adopting names was irregular. He would not object to the resolution without section 1.

Dr. Latham asked the meeting to accept Colonel Smith's amendment in preference to the preceding one. This was agreed to, and was likewise carried on being put as a substantive motion.

Mr. W. Bishop Harman thought this meeting should not try to override the expressed wishes of 23,000 members of the British Medical Association, especially as during the past six months the Committee of that Association had accumulated a great deal of valuable experience.

Sir Thomas Horder moved: "That the Representative Committee consist of (i) a representative of each of the following ten organisations or corporations: 1. Royal College of Physicians; 2. Royal College of Surgeons; 3. Royal Society of Medicine; 4. British Medical Association; 5. Incorporated Society of Medical Officers of Health; 6. Medical Society of London; 7. Medical Political Union; 8. Medical Women's Federation; 9. Poor-Law Medical Officers' Association; 10. State Medical Service Association. (ii) Unofficial members.

The result of this was a decision to appoint a Committee and to include among the bodies represented the Royal Army Medical Corps. A plea for representatives of the insane brought the Chairman's retort that rectal surgery was not specially represented either.

Dr. Chapple, M.P., led a vigorous protest against the proposal that "persons who do not hand in voting papers will be counted as having voted for the list as printed," and it was dropped. Voting papers were deposited, and a vote of thanks to the Chairman and Dr. Addison closed a long and strenuous meeting.

